# Investigation of the presence of human or bovine respiratory syncytial virus in the lungs of mink (*Neovison vison*) with hemorrhagic pneumonia due to *Pseudomonas aeruginosa*

**DOI:** 10.1186/1751-0147-54-70

**Published:** 2012-11-26

**Authors:** Charlotte M Salomonsen, SolvejØ Breum, Lars E Larsen, Jeanette Jakobsen, Niels Høiby, Anne S Hammer

**Affiliations:** 1 National Veterinary Institute, Technical University of Denmark, Bülowsvej 27, DK-1870, Frederiksberg C, Denmark; 2 Department of Clinical Microbiology, Rigshospitalet, Blegdamsvej 9, DK-2100, Copenhagen Ø, Denmark; 3 Department of Veterinary Disease Biology, Faculty of Health and Medical Sciences, University of Copenhagen, Ridebanevej 3, DK-1870, Frederiksberg C, Denmark

**Keywords:** Hemorrhagic pneumonia, Mink, *Pseudomonas aeruginosa*, Respiratory syncytial virus

## Abstract

**Background:**

Hemorrhagic pneumonia is a disease of farmed mink (*Neovison vison*) caused by *Pseudomonas aeruginosa*. The disease is highly seasonal in Danish mink with outbreaks occurring almost exclusively in the autumn. Human respiratory syncytial virus (RSV) has been shown to augment infection with *P. aeruginosa* in mice and to promote adhesion of *P. aeruginosa* to human respiratory cells.

**Findings:**

We tested 50 lung specimens from mink with hemorrhagic pneumonia for bovine RSV by reverse transcriptase polymerase chain reaction (PCR) and for human RSV by a commercial real-time PCR. RSV was not found.

**Conclusions:**

This study indicates that human and bovine RSV is not a major co-factor for development of hemorrhagic pneumonia in Danish mink.

## Findings

*Pseudomonas aeruginosa* was identified as the cause of hemorrhagic pneumonia in farmed mink (*Neovison vison*) in 1953 [1]. This disease can cause mortalities as high as 50% on the farm level [2,3] but can also follow a milder course [1]. Hemorrhagic pneumonia is characterized by a rapid disease progression in the individual mink and it is highly seasonal in Denmark with farm outbreaks occurring almost exclusively from September to November. Whether the seasonal pattern is related to the seasonal life cycle of farmed mink or if it relates to co-infection with other pathogens has not been investigated. Thus, no infectious co-factors have been identified but the highly seasonal appearance of disease may indicate the presence of a viral co-factor. In human cystic fibrosis (CF) patients, a seasonal pattern for acute and chronic *P. aeruginosa* infection has been identified [4] and the authors proposed respiratory viruses as the underlying cause for this pattern. Respiratory viruses have a seasonal infection pattern coinciding with that of *P. aeruginosa* infection in CF patients and respiratory viruses are known to precede bacterial infections [5,6].

Respiratory syncytial virus (RSV) is an enveloped single-stranded negative sense RNA virus belonging to the genus *Pneumovirus* in the family Paramyxoviridae. Variants of the virus infect various species including sheep, goats, mice, cattle and humans. A number of animal species can be experimentally infected with human RSV (HRSV) [7,8]. The occurrence of HRSV infections in humans is known to be highly seasonal with the majority of cases occurring in the winter or spring [9]. HRSV has been shown to promote adhesion of *P. aeruginosa* to cells in the respiratory tract of mice [10,11] and to directly mediate binding of *P. aeruginosa* to human epithelial cells *in vitro*[12]. HRSV has also been shown to replicate in mink lung cells *in vitro*[13] and in the lungs of infant ferrets [14]. RSV has never been described as naturally occurring in mink and experimental studies on RSV infections in mink has not been published. However mink may be exposed to HRSV by their human caretakers and to bovine RSV (BRSV) from the feed which often include slaughter offal from cattle.

The aim of the present study was to investigate whether HRSV or BRSV could be found in lung tissue from mink succumbing to hemorrhagic pneumonia caused by *P. aeruginosa* and hence possibly be part of the pathogenesis of this disease.

Fifty samples of lung tissue from Danish mink were selected of which 40 originated from mink submitted from 10 fur farms for routine diagnostic examination and found to have hemorrhagic pneumonia associated with culturing of *P. aeruginosa* in pure culture from the lungs. Twenty of these mink originated from three farms with high mortality due to hemorrhagic pneumonia (above 15%) while 15 mink were submitted from five farms with low mortality (below 2%). Data on mortality was not available for the last five mink submitted for routine diagnostic investigation. Ten samples originated from normal wildmink experimentally infected intra-nasally with 10^3^-10^9^ viable *P. aeruginosa* bacteria. Two of the ten experimentally infected mink developed clinical symptoms of hemorrhagic pneumonia (lethargy, labored breathing) and were euthanized two days after inoculation and necropsied immediately. The remaining eight clinically normal mink were euthanized eight days after inoculation and necropsied immediately. These eight mink served as negative controls. The animal experiments were approved by the Danish Animal Experiments Inspectorate. From the control mink, specimens of the left caudal lobe was obtained, while lung tissue with gross lesions of pneumonia was chosen from the experimentally infected clinically diseased mink and the mink submitted for routine diagnostic. The samples were collected during 2011 and stored at −20°C until further testing.

Total RNA was purified from the lung tissue using the RNeasy Mini Kit (Qiagen, Düsseldorf, Germany) on a QIAcube (Qiagen) according to manufacturer’s instructions. A BRSV positive cell culture was used for positive control while nuclease-free water (Amresco, Solon, OH, USA) was used as a negative control of the purification step. The recovered RNA was stored at −80°C until further analysis.

The presence of BRSV was tested with a modification of a previously published conventional reverse transcriptase polymerase chain reaction (RT-PCR) assay targeting the F-gene with the forward primer sequence 5^′^-AACCGGCTTCCTTCAGTAGAGC-3^′^ and the reverse primer sequence 5′-CAATACCACCCACGATCTGTCC-3^′^[15]. The RT-PCR was performed on a Biometra T3 Thermocycler (Biometra, Goettingen, Germany) in a total volume of 25 μl using Qiagen OneStep RT-PCR kit (Qiagen) and 3 μl of extracted RNA, 400 μM dNTP mix and 500 nM of each primer. The amplification temperature profile was 30 min at 50°C for reverse transcription, 15 min at 95°C and 35 cycles of 30 s at 95°C, 30 s at 55°C and 1 min at 72°C, followed by 10 min at 72°C. In each PCR reaction a known BRSV positive sample and a non-template control was run with the samples as controls. PCR products with the expected size of 730 bp were visualized on E-gel® 2% General Purpose Agarose (Invitrogen, Carlsbad, CA, USA).

For detection of HRSV a commercially available real-time PCR (Prodesse® ProFlu™+, Gen-probe, San Diego, CA, USA) was employed. The primers used for this detection were not available due to the commercial nature of the PCR. The amplification and detection were performed on a MX3005p (Stratagene, La Jolla, CA, USA). The manufacturer’s protocol was followed in detail.

The 50 mink lung samples were negative when tested in the BRSV and HRSV specific assays. In 5 samples tested with the BRSV specific primers, weak PCR products of the predicted size were recognized but sequence analysis showed no resemblance to the F gene of BRSV or to other known genes for these products. Therefore, they probably represent unspecific annealing to mink specific nucleic acids. No PCR products were obtained using the HRSV specific assay.

Evidence of concurrent infection with HRSV and BRSV and *P. aeruginosa* in cases of mink hemorrhagic pneumonia was not found. Whether this was due to breakdown of the virus, e.g. due to severe inflammation, collection of samples after clearance of the virus, or because HRSV or BRSV simply was not present in the lung samples remains uncertain.

Histological examinations of lungs from mink suffering from hemorrhagic pneumonia have not revealed syncytial cells [16,17] but due to the severe lung lesions seen in this type of infection, syncytial cells may be difficult to recognize or may be necrotic at the time of histological examination. RSV can experimentally infect a number of species [8] but is apparently quite host-specific in eliciting disease even though the various RSVs are closely related [7]. If a specific mink RSV is existing and is associated with hemorrhagic pneumonia in mink, the primers used for detection of BRSV and HRSV in this study may not be able to detect this virus. To further elucidate whether a novel RSV-like virus is present in mink, other primer-sets may be generated based on highly conserved parts of genomes belonging to the genus *Pneumovirus*. Electron microscopy, immunohistochemistry of lungs and nasal epithelium or *de novo* sequencing of tissue from animals showing acute respiratory symptoms may also be a valuable tool for identifying unrecognized respiratory viruses that may act as co-factors to *P. aeruginosa* hemorrhagic pneumonia.

## Competing interests

The authors declare that they have no competing interests.

## Authors’ contributions

CMS participated in the design of the study, identified and collected suitable material, coordinated the study and drafted the manuscript. SØB participated in the design of the study, supervised the BRSV testing, interpreted the sequencing of the weak PCR products and revised the manuscript. LEL contributed to the study design and revised the manuscript. JJ planned the HRSV testing and performed the practical experiment. NH contributed to the study design. ASH participated in the design of the study and the initial identification of tissue used in the study. All authors read and approved the final manuscript.
